# Dynamic shapes of the zygote and two-cell mouse and human

**DOI:** 10.1242/bio.059013

**Published:** 2021-12-22

**Authors:** Chris F. Graham, Shane Windsor, Anna Ajduk, Thanh Trinh, Anna Vincent, Celine Jones, Kevin Coward, Dilraj Kalsi, Magdalena Zernicka-Goetz, Karl Swann, Adrian L. R. Thomas

**Affiliations:** 1 Zoology Department, University of Oxford, 11a Mansfield Road, Oxford, OX1 3SZ, UK; 2 Nuffield Department of Women's Reproductive Health, Level 3, Women's Centre, John Radcliffe Hospital, Oxford, OX3 9DU, UK; 3 Department of Aerospace Engineering, University of Bristol, Queens Building, University Walk, Bristol, BS8 1TR, UK; 4 Department of Embryology, Faculty of Biology, University of Warsaw, Miecznikowa 1, 02-096 Warsaw, POLAND; 5 Cleveland Clinic Fertility Center, 26900 Cedar Rd., Beachwood, OH 44122, USA; 6 Oxford Fertility, Oxford University, Oxford Business Park North, Alec Issigonis Way, Oxford, OX4 2HW, UK; 7 Nuffield Department of Surgical Sciences, University of Oxford, Oxford, OX3 9D, UK; 8 Department of Physiology, Development and Neuroscience, Downing Street, Cambridge, CB23 3EL, UK; 9 School of Biosciences, Sir Martin Evans Building, Museum Avenue, Cardiff, CF10 3AX, UK

**Keywords:** Mouse zygote, Morphokinetics, Shape cycles, Pronuclear fading, Human, Cytoplasm vortices

## Abstract

Mouse zygote morphokinetics were measured during interphase, the mitotic period, cytokinesis, and two-cell stage. Sequences of rounder–distorted–rounder shapes were revealed, as were changing patterns of cross section area. A calcium chelator and an actin-disrupting agent inhibited the area changes that occurred between pronuclear envelope breakdown and cytokinesis. During cell division, two vortices developed in each nascent cell and they rotated in opposite directions at each end of the cell, a pattern that sometimes persisted for up to 10 h. Exchange with the environment may have been promoted by these shape and area cycles and persisting circulation in the cytoplasm may have a similar function between a cell's interior and periphery. Some of these movements were sporadically also seen in human zygotes with abnormal numbers of pronuclei and the two-cell stages that developed from these compromised human zygotes.

## Abstract (precis)

**Summary:** Mouse zygote morphokinetics were measured during interphase, the mitotic period, cytokinesis, and two-cell stage. Sequences of rounder–distorted–rounder shapes were revealed, as were changing patterns of cross section area. Some of these movements were sporadically also seen in human zygotes with abnormal numbers of pronuclei and the two-cell stages that developed from these compromised human zygotes.

## INTRODUCTION

The purpose of the present paper was to search for movements of the zygote and two-cell stage that might increase exchange between the embryo and its environment in culture (see Discussion).

The emphasis is on the changing space between the cell membrane and acellular matrix of the zona pellucida (ZP) because alterations here give strong flow in a closed hydraulic system. The disposition of this space was already known to alter in the zygote and two-cell stage (Ajduk et al., 2011; Deguchi et al., 2000; Gardner and Davies, 2003; Kurotaki et al., 2007; Waksmundzka et al., 1984). In the present study, additional movements were found, measured, and analysed during zygote interphase, pronuclear envelope breakdown (PNEBD), the mitotic period, cytokinesis, and the two-cell stage. Similar shape changes were seen in some human embryos that developed from zygotes with abnormal numbers of pronuclei (Supplementary Material). Inside each cell of the two-cell stage bulk cytoplasm flows were noticed and these flows differed from most movements of cytokinesis in that there were vortices at both ends of each nascent cell, and vorticity sometimes persisted for up to 10 h into the two-cell stage, invertebrate cytokinesis reviewed (Rappaport, 1996).

## RESULTS

Zygotes were obtained from the mouse crosses CD1xCD1 and F1C57BL6/CBAxF1C57BL6/CBA (the latter called F2 embryos). This study only included those early stages that subsequently developed into morulae and blastocysts by 96 h post the superovulation injection of human chorionic gonadotrophin (96 hpHCG). The main methods for measuring and analysing cell shape and cytoplasm movement were the development of bespoke software (cell_piv) that outlined the zygote membrane, calculated the centre of area (CoA), and the length of 36 radii and 18 diameters at 10° of rotation around CoA (see Materials and Methods).

**Fig. 1.**
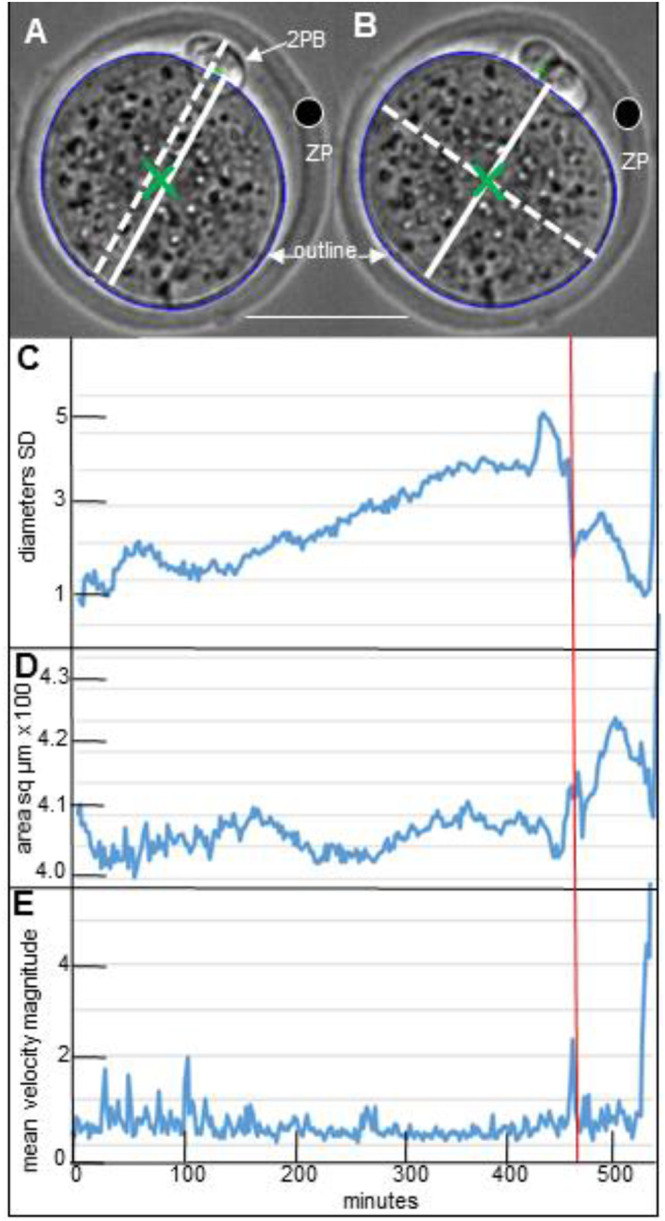
**The major changes of diameter variation and cross sectional area up to elongation in division.** (A,B) Cell outline (blue), centre of area (CoA, large green cross), second polar body (2PB), and a granule in ZP (large outlined black dot). A black dot was superimposed on a pale irregularity in the ZP. (A) All diameters passed through the CoA. The zygote diameter based on the 2PB (solid white line), while this diameter plus the width of the 2PB is the broken white line (2PB diameter+2PB). (B) The 2PB zygote diameter (solid white line), and its perpendicular (broken white line). Elongation was seen in B photographed 20 mins after A. The scale bar indicated c.50 µm. (C-E) The vertical red line marks the frame at which PNEBD was observed, that is the time when the last large nucleolus disappeared from remnants of the pronuclei. Panels C and D were derived from the outline around the zygote and the information refers to the single focal plane of maximum zygote cross section area with a protruding 2PB. (C) The standard deviation (SD) of the 18 diameter lengths around CoA at every 10° of rotation gave an indication of shape. (D) Area was measured within the outline. (E) The mean magnitude of movement was the particle image velocimetry (PIV) measured in a rectangle inside the cytoplasm. As applied here and elsewhere, PIV measured the change of velocity between two sequential frames, see Ajduk et al., 2011.

The ZP and zygote were known to be asymmetric in three dimensions and the longest ZP diameter was the 2PB+2PB diameter, a slightly shorter diameter was at right angles to it, and the shortest diameter of all was on or parallel to the z axis through CoA when the zygote was in the standard position (Fig. 1A) (Alarcón and Marikawa, 2008; Gardner, 1997).

**Fig. 2.**
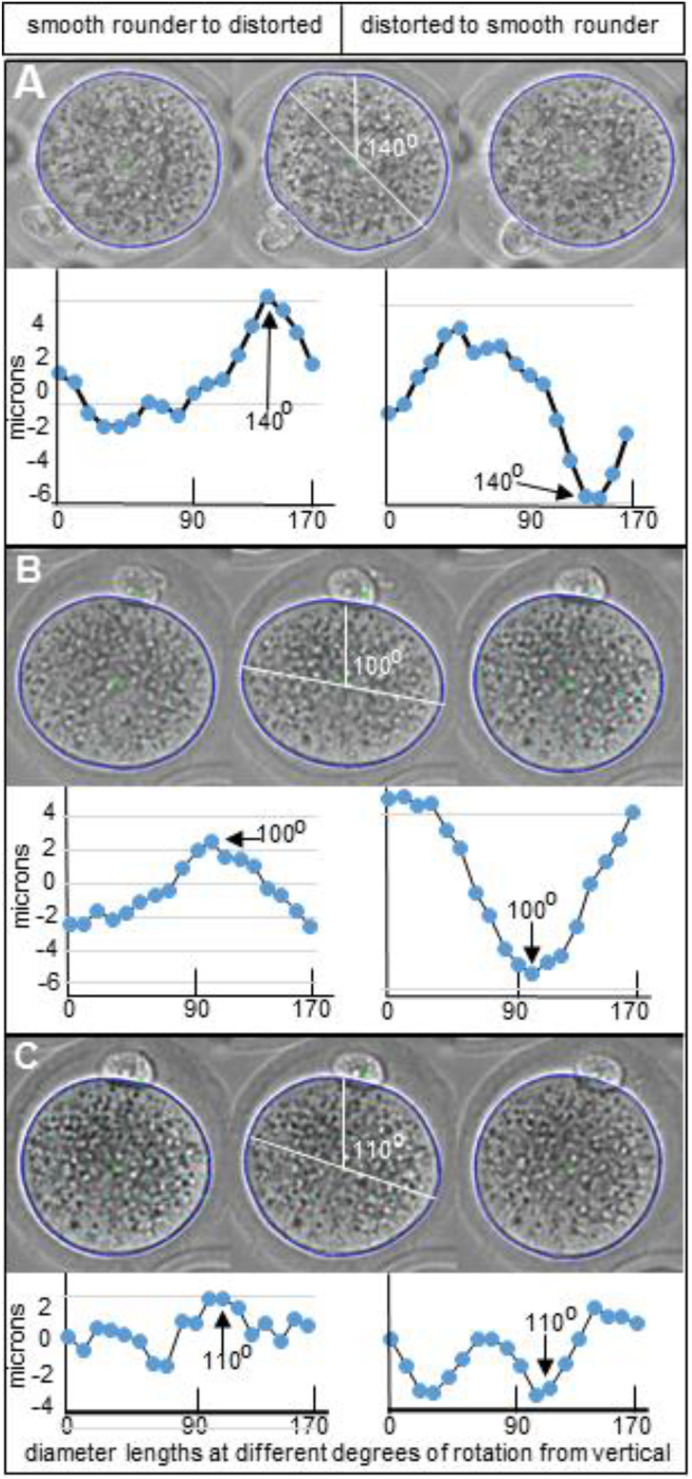
**Sequences of shape changes at different periods of zygote development.** At all periods, the zygote images displayed the sequence rounder–distorted–rounder shapes, and the charts below the images plotted the change of diameter lengths between the left and middle image and between the middle and right image. The vertical diameter was at 0° rotation and the diameter changes at each of the 10^o^ of clockwise rotation were shown as pale blue dots. (A) An Interphase sequence. During 82 mins, there was a notable extension (nose) of the 140° of rotation diameter (white line on zygote image), and then over the next 58 mins the zygote rounded up and this diameter shortened. (B) A PNEBD sequence. During 18 mins, the ovoid shape was emphasised by an extension of the 100° diameter. Rounding up of this ovoid shape during another 18 mins involved both diameter extension around the 2PB as well as shortening of the 100° diameter. (C) A mitotic sequence in the same zygote as B. During 24 mins the zygote extended at 100-120° of rotation, and then rounded up again during the following 68 mins. The greatest extension–retraction changes during this sequence were at 110° rotation. Additional details of mouse and human sequences are in Fig. S1.

Asymmetry in two of the previously described ZP dimensions was confirmed in CD1 zygotes of different ages. In the position shown in Fig. 1A, the ZP was shown to be slightly oval during interphase, so that when its diameter through CoA and the protruding 2PB was divided by its perpendicular, then the mean ratios were greater than 1.0, and this was also the longest ZP diameter (1.093 at 13.5-16.5 hpHCG; 1.058 at 24-27 hpHCG, *n*=10 CD1). The corresponding diameter ratios in the zygote including the 2PB were 1.103 and 1.094 at these times. The ZP had ‘hardened’ before the present study period, and the ZP retained its shape after the zygote has been removed (Gardner and Davies, 2006; Khalilian et al., 2010; Krzanowska, 1972). The ZP's was also known to vary between and within mouse strains with respect to its resistance to proteases and its mechanical properties (Krzanowska, 1972; Yanez et al., 2016). The conclusion was that an asymmetric zygote displayed shape changes inside a robust asymmetric matrix under the conditions of the present work (Fig. 2).

### Common features of zygote shape sequences

The previous reports of sudden rounding up at the start of the mitotic period were supplemented by the observation that similar movements occurred in sequences, which began during interphase and sometimes lasted up to cytokinesis. The zygote profile varied between a distorted and a rounder shape (Fig. 2). A convenient way to detect the shape sequences was to plot the standard deviation of the 18 diameters in each frame against time and these showed irregular peaks and troughs (SD, Fig. 1C): the diameters were measured at the high and low points of these plots (Fig. 2). These measurements defined a shape sequence for the purposes of this paper: the first half of the sequence had to involve at least a 1.5 µm extension along at least one diameter, followed by an abbreviation of the same diameter in the second half of the sequence (Fig. 2A-C). The diameter with the maximum in-out-in change in a sequence ranged from 2.7 µm to 14.8 µm at different stages of zygote development (Table 1, column 3).

**Table 1.**
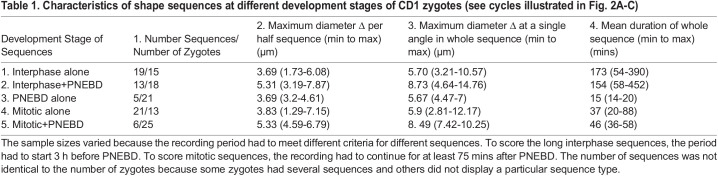
Characteristics of shape sequences at different development stages of CD1 zygotes (see cycles illustrated in Fig. 2A-C)

### Stage-specific sequences

Shape sequence characteristics and numbers differed as the one-cell stage progressed (Table 1, Fig. 2). Isolated interphase cycles occurred in the 15/18 CD1 zygotes, and in most of these zygotes, there were additional interphase sequences that overlapped with PNEBD (Fig. 1C). F2 zygotes had reduced shape changes (not shown). A few PNEBD sequences did not grossly overlap with interphase sequences in CD1 zygotes, but even in these it was difficult to isolate the PNEBD shape changes from those of the preceding interphase sequence or the following mitotic sequence. In all situations, PNEBD shape sequences coincided with or were nearly in time with sharp increases of cytoplasmic speed as measured by particle image velocimetry (PIV; Fig. 1E). Although the numbers were small, the PNEBD shape sequences were clearly short (Table 1, row 3; F2, data not shown). Mitotic sequences occurred in 13/25 of CD1 zygotes: in five of these zygotes, the PNEBD event was combined with the mitotic changes into a single sequence (Table 1, rows 2 and 3). F2 and MF1 data was similar (not shown). Given that the asymmetries of the ZP were retained during these events, it was concluded that some regions of the zygote membrane substantially altered their distance from the ZP within each sequence (Table 1, column 3 and example in Figs 2 and 5).

### Area changes

**Fig. 3.**
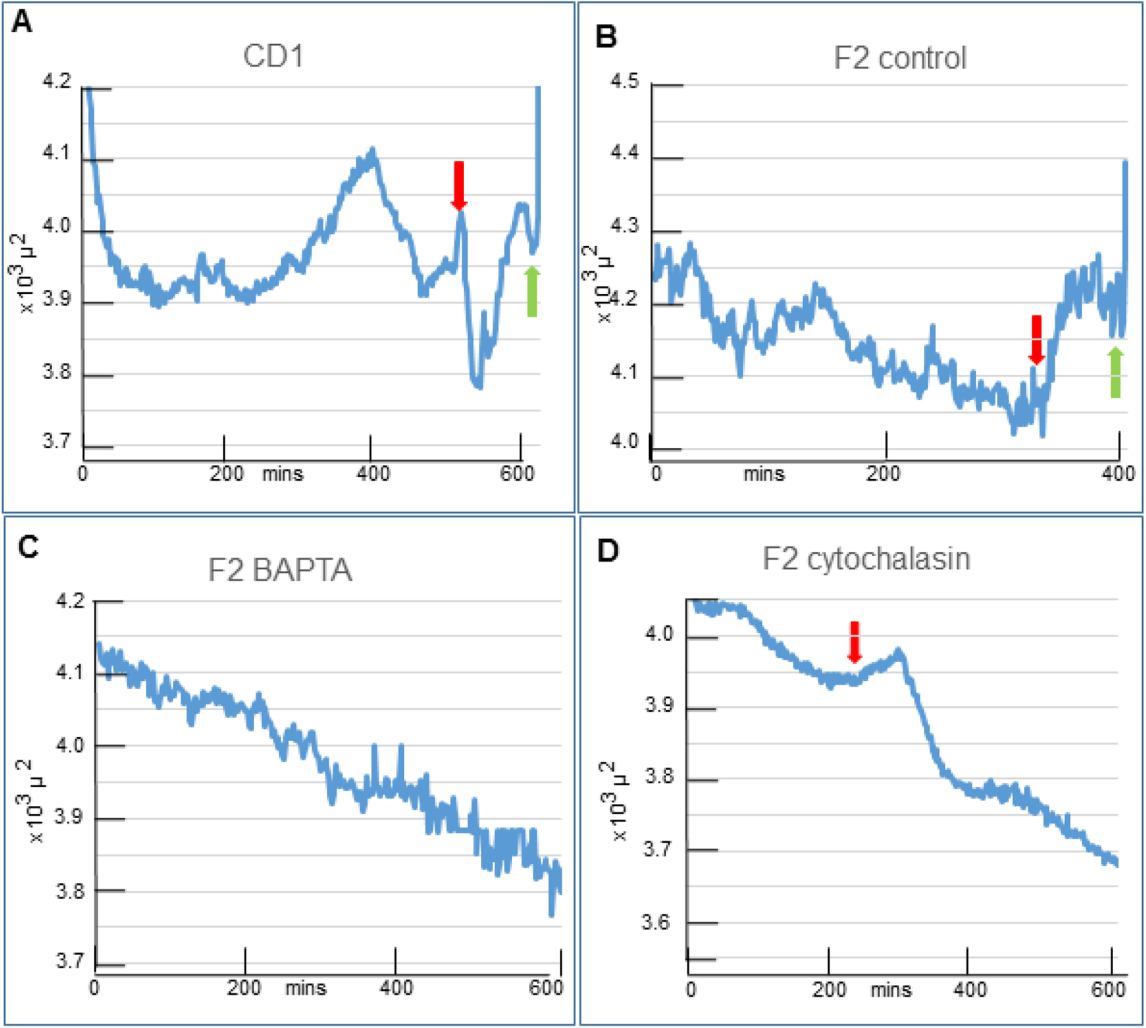
**Area changes and inhibitor actions.** The range of area values was 500 µm^2^ for the y axis in each graph but the starting point of the y axis varied between the graph panels. A down red arrow pointed to the frame when PNEBD was percieved and the green upward arrow pointed to the frame when rapid zygote elongation began (charts A,B).

The changes in average cross section area followed a pattern in most control CD1 and F2 zygotes, and these were relatively minor alterations involving 7-9% of the maximum zygote area changes during the recording period (Fig. 3A,B). Beginning near the start of recording, a common pattern was that the areas declined, and reached and maintained low values until the areas rose slightly before PNEBD, and then rose again to reach a peak before falling as the zygotes rounded up just before the elongation of cytokinesis. In detail, for 17 CD1 zygotes, 14 underwent PNEBD at low cross section area values. After PNEBD, the area quickly reduced, and this was usually the lowest value before cytokinesis (quick reduction interval: mean 19 mins, range 2-50 mins). Despite wide variation in this pattern between zygotes, it was clear that these changes in cross-sectional area altered the sub-zona space in the observation plane. However, in the absence of data in the third dimension it was not known if these area changes altered the total volume of sub-zona space or merely its disposition.

### Area changes depended on Ca2+ ions

We explored the action of inhibitors at identical concentrations to those with established effects on the zygote's cytoskeleton (Ajduk et al., 2011). It was known that calcium transients frequently coincided with PNEBD and in some studies they continued into the post-PNEBD period of mitosis (Day et al., 2000; Kono et al., 1996; Larman et al., 2004; Marangos et al., 2003; Tombes et al., 1992). F2 zygotes were used to examine the action of inhibitors on the common pattern of area changes because the association between area changes and the PNEBD events were relatively uniform on this background (Fig. 3B). When the zygotes were in a similar position to those in Fig. 1A, 16 of 18 exhibited PNEBD at low area values (Fig. 3B). The brief increase in area before division was seen in 14 of 18 F2 zygotes.

It was possible that these area changes depended on the Ca2+ transients and this idea was tested by treating the zygotes with BAPTA-AM, a Ca2+ chelator. The chelator inhibited PNEBD and division and in these zygotes the area declined progressively throughout the recording period (10/11 cases) (Fig. 3C), and the conclusion was that the mitotic area rise depended on Ca2+. The variation of area in the BAPTA treated group amounted to 10% of the maximum area recorded in this group (*n*=11).

An attempt was made to study the influence of reduced Ca2+ on shape sequences in the zygotes which lacked PNEBD and whose areas changes had been inhibited by BAPTA. Small-scale shape sequences just above background noise were found and these lasted for a variety of periods, but the known overlap between the lengths of sequences at different times of the one-cell stage (Table 1) made it impossible to decide which if any of the shape sequences had been inhibited.

### Area changes depended on an intact actin cytoskeleton

The extent of area changes also depended on the actin cytoskeleton. PNEBD occurred in 24 out of 27 F2 zygotes treated with cytochalasin D, an actin depolymerizing agent. Again, area declined progressively but there was a brief interruption around the time of PNEBD (Fig. 3D), when there was a temporary rise in the cross-sectional area (19/24). In the remaining five, the area decline was briefly interrupted. In six out of 19 zygotes, a surface dimple was seen during this brief area expansion and as the dimple began to regress so bodies with the appearance of nucleoli were found close to the dimple (Fig. S2). Later these bodies were included into nuclei that moved towards the centre of the zygote: it was assumed that similar events occurred out of the image plane in the remaining 13 of the 19 zygotes. This result showed that this local shape and area change depended on events that differed from those at the fertilization cone (FC) which were suppressed by cytochalasin (Ajduk et al., 2011; Toth et al., 2006), see also references in (Deng and Li, 2009). The variation of area in the cytochalasin treated group amounted to 14% of the maximum area recorded in this group (*n*=23).

### Cytokinesis: elongation and slimming

**Fig. 4.**
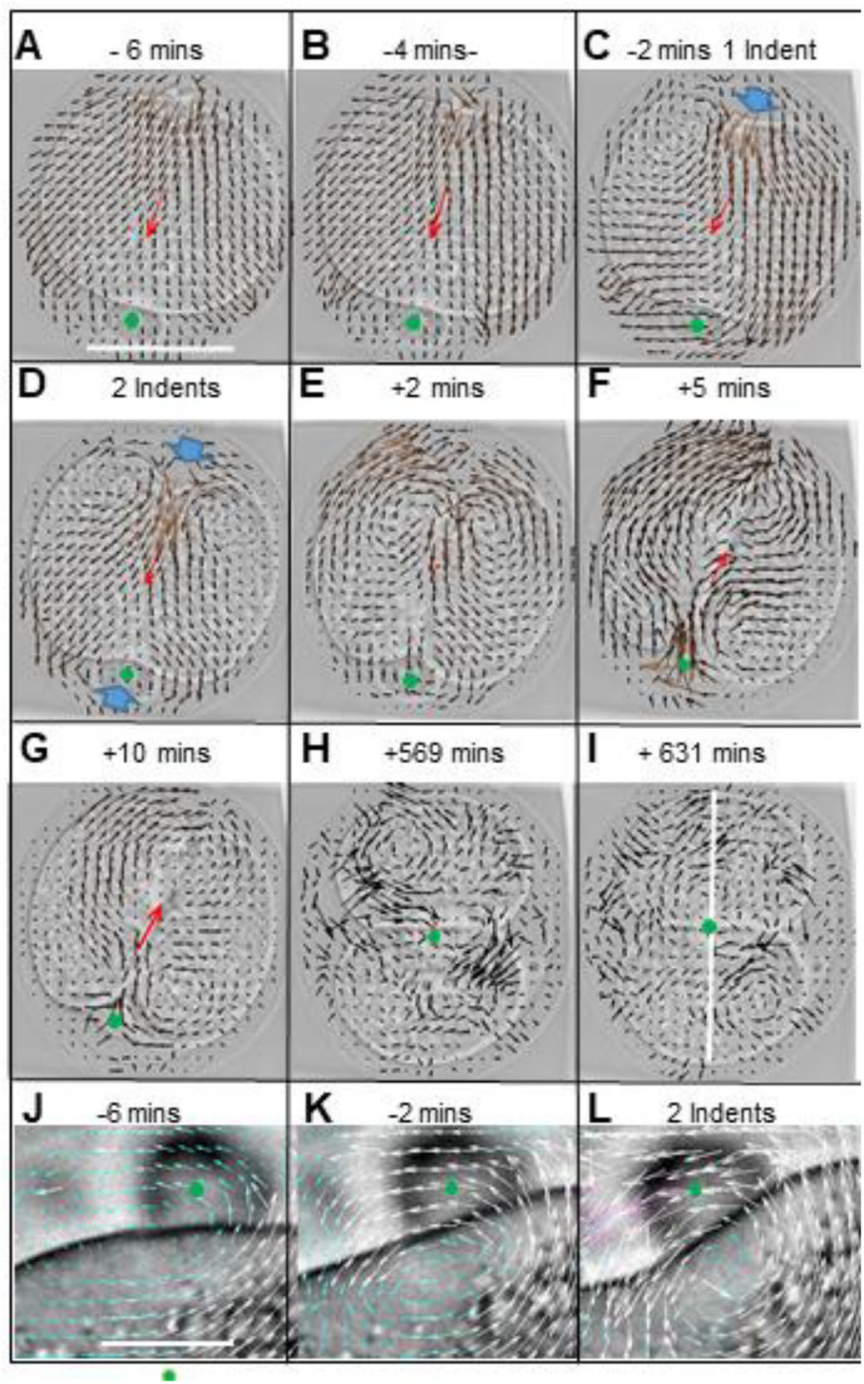
**Cell division and two-cell stage cytoplasm flow.** (A-I) The time was shown in relation to the two-indents stage (scale bar in A: 50 µm, vector arrow direction and magnitude reported flow in c. 4.7 µm^2^). The near vertical white line in I shows the two-cell axis. The vectors are dark arrows, the mean vector is a red arrow, and the centre of the 2PB is a green dot. Vortices were visible in the pattern of vectors (see text). The one-indent and two-indents stages display blue fat arrows that draw attention to the indents on the zygote. Images collected every 1 min. (J-L) Cytoplasm motions in a second embryo were shown near the 2PB (scale bar *c*. 14 µm, each vector arrow direction and length reported flow in *c*. 2 µm^2^). To make them visible, the vectors are magnified x10 compared to those in panels A-I and displayed as white arrows. Image capture every 2 mins.

The present data confirms the set pattern of shape changes of cytokinesis that have been described (Duch et al., 2020). Sub-zona space distribution changed rapidly. The angle and extent of zygote elongation in division was measured after first finding the diameter with the greatest extension between an earlier time and the times when the first furrow of cytokinesis was observed on the zygote surface, the one-indent stage illustrated in Fig. 4C. The ‘first step’ of rapid elongation was taken to be the first time when the elongation diameter increased by 1 µm or more in a 2 mins interval, and it was an early event because 22 of 25 ‘first steps’ occurred about 4 mins before the first indent was seen (Fig. 4A). The zygotes and surrounding ZPs continued to elongate after this one-indent stage. The one-indent stage was followed about 2 mins later by the two-indents stage when a second indent formed on the opposite side of the zygote (Fig. 4D).

**Fig. 5.**
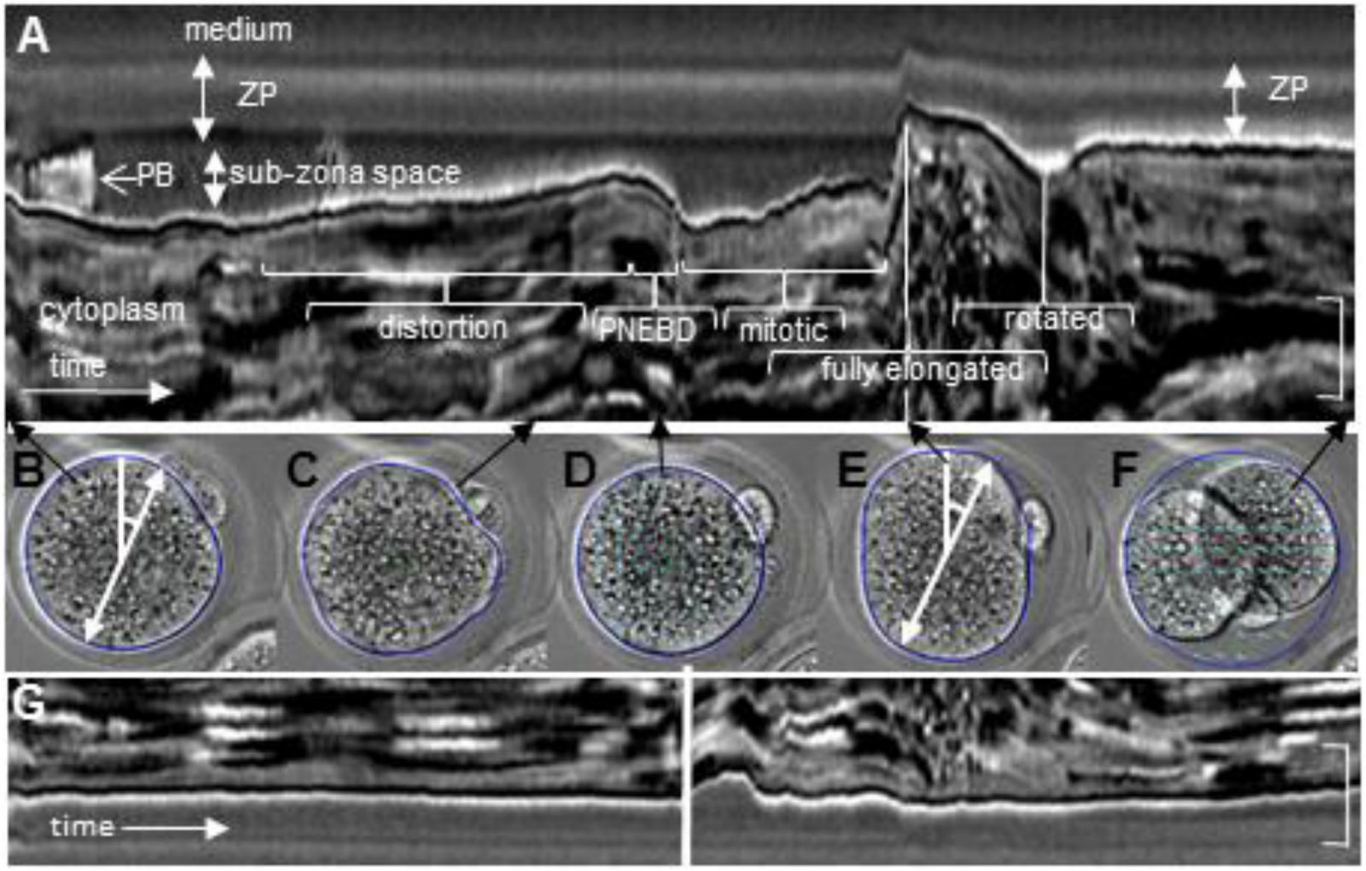
**Summary of mouse zygote movements.** This record runs for 620 mins at 2 mins interval image collection. For kymograph (A) the coincident whole zygote images were displayed below, and for (B and E) a diameter at 20° rotation from the vertical was set out (broad white line with arrows at both ends). The cytoplasm movements were shown across about *c*. 50 µm of this line. A darker band in the ZP was the trail of a dark granule in the ZP. The zygote surface distorted (B and C) and smoothed over around the time of PNEBD as the rounder form developed. The zygote surface was pushed out at maximum cell elongation (E). Soon after, the oblique two-cell axis had swung round and nearly aligned with the previous position of the 2PB diameter (F). During the E to F transition, the 2PB disappeared from the cell profile (not shown) only to reappear again (F). Note also that the ZP has retained its position as indicated by the dark band in the ZP while 2PB has changed its position with respect to the granule. (G) Kymograph recorded at the bottom end of the 20° diameter and the cell membrane lay against the ZP for most of the recording. The zygote indented and shortened at both ends of the diameter at PNEBD. In contrast, at elongation E, the extension was at the top of this diameter where initially there was sub-zona space available. This kymograph was scored as having one interphase+PNEBD sequence and one mitotic sequence.

In 18 zygotes, elongation lasted 10 mins (range 4-17 mins) and its elongation axis (diameter) extended by 11.6 µm (range 4-22 µm). The poles of the elongation axis usually touched and flattened against the ZP, and during elongation the ZP was extended along a similar axis (Fig. 5A and E). In 18 out of 19 CD1 zygotes, there was a mean ZP extension of 2.69 µm, range 0.81 to 6.84 µm: the exceptional ZP shrunk by 1.85 µm along this axis. The elongating CD1 zygotes also narrowed so that the ratio of elongation diameter length divided by its perpendicular through CoA was greater than 1. When both the ‘first step’ and first indent occurred in focus, the ratios were 1.07 at first step and 1.25 at one-indent (*n*=12). The ZP also narrowed perpendicular to the angle of elongation (in 14/18 zygotes, by a mean of 1.56 µm, range 0.65 to 3.14 µm, the exceptional four zygotes showed a mean ZP increase of 1.12 µm, range 0.25 to 2.37 µm). Despite the coordinated extension and slimming of both the zygote and the ZP, the observation was that sub-zona space increased and decreased in limited regions during this period of division (Fig. 5).

### Pitch, rotation, and roll of the whole emerging two-cell stage

The zygote and two-cell stage pitched, rotated and probably rolled inside a static ZP. These acrobatics substantially rearranged the sub-zona space during a mean period of 8 h after division, while the asymmetries of the zona and embryo were retained: the ratio of the two-cell axis to its perpendicular axis was 1.10 for the embryo and 1.048 for the ZP. Consequently, these manoeuvers were interpreted as interactions between the asymmetric ZP and both the asymmetric elongating zygote and the asymmetric two-cell stage (see Fig. S3 for mouse and human examples).

### Pitching

The evidence for pitching was provided by the behaviour of the elongation axis in the x–y plane. As this extended to its maximum length, so one end moved out of focus (28/40 CD1 and F2 zygotes), and this tilt usually began 2 mins before the one-indent stage. As the daughter cells’ perimeters became clear, so their outlines overlapped, and division appeared to be oblique to the original axis of elongation (Fig. 5F; Fig. S3).

### Rotating

It was seen that the two-cell boundary rotated in the x–y plane (Figs 4G,H and 5E,F), and the purpose of this section was to measure the extent and speed of the rotation, a phenomenon that had first been noticed by others (Kurotaki et al., 2007). The rotation was first seen by movements of the indent diameters, the line from the first indent through CoA across to the other side of the zygote or between the two indents when both were visible. About one third of the total rotation 5 h later had occurred by 24 mins after the two-indents stage (*n*=14, range 10-79%, Fig. 5E,F) and in the first hour, 45% of the 5-h total had been achieved. Slower rotation continued for up to 10 h (*n*=7). To summarize, during the recording periods rotation started during cytokinesis and was concentrated in the first hour after the two-indents stage. These observations do not exclude further rotation at later stages of development.

### Rolling

The evidence for rolling after division came from studying the position of the 2PB. The assumption was that once the 2PB had reached the groove of the cytokinetic furrow then it stayed in the furrow and did not rotate around that furrow. In 26 CD1 and F2 zygotes, the 2PB started at least one third proud of the zygote profile and during or soon after division it disappeared into the two-cell image (Fig. 4G,H). The 2PB remained obscured throughout the recording in 12 of these zygotes. In the remaining 14 zygotes, the 2PB reappeared 2/3 proud of the two-cell profile 235 mins later (range 70-882 mins) where it stayed in place until recording ended 77 mins later (range 0-168 mins). This peek-a-boo behaviour of the 2PB was interpreted as the two-cell stage rolling around the two-cell axis (Fig. 4I), possibly fitting its shortest diameter into the previous shortest diameter of the one-cell stage ZP. The conclusion was that the nascent and fully formed two-cell stage displayed a rich range of movements inside a static matrix.

### The internal circulation of the cytoplasm

The purpose was to find out if cytoplasm circulation was available to promote exchange between the internal cytoplasm and the cell periphery. The result was that unusual patterns of bulk cytoplasm motion were found and these sometimes continued for 10 h after division.

### Cytokinesis

Cytoplasm flow during division was first analysed at low resolution by calculating a mean vector that represented the arithmetic mean of all the movement vectors inside the computed cell outline (red arrow in Fig. 4A-G). The majority of mean vectors pointed towards the 2PB half of the zygote at the first step of elongation (15/22), but by the one-indent stage 12/22 mean vectors pointed away from the half with 2PB at its centre as the second indent started to form. Sometimes this reversal of the mean vector direction was observed after the two-indents stage (Fig. 4F-G).

The pattern of vectors gave a finer grained analysis of cytoplasmic motion. The future position of an indent was anticipated by a localized inflow before the indent itself was seen (Fig. 4A,B). As this first inflow progressed, so vortices developed in the nearby cytoplasm of each emerging cell (Fig. 4D,E). Opposite and near the 2PB, a cell surface depression began to deepen 2 mins before the one-indent stage. For a short time, this movement was not accompanied by significant cytoplasm inflow and it may have been a consequence of the 2PB pressing into the zygote as the ZP narrowed during elongation (Fig. 4C,D). Later and sometimes starting as early as the one-indent stage, both the change of shape and cytoplasm flow coincided near the 2PB (Fig. 4F). And near this second inflow region another pair of vortices formed (Fig. 4G).

The origin of vectors was clearest near the indent closest to the 2PB: these were examined at higher vector density (Fig. 4J-L). It was observed that the base of the 2PB was moving in the same direction as cell components very close to or at the cell surface and measurements of the 2PB moves confirmed previous work (Bauer et al., 2008). Slightly deeper cytoplasm consistently flowed outwards on either side of the 2PB as it appeared to ski down into the deepening groove of the future boundary between the cells of the two-cell stage (Fig. 4L). The conclusion was that the vortex in the cytoplasm at the 2PB end of the cell originated from the counter moves of cytoplasm moving into the cytokinetic furrow and cytoplasm further away rising up on either side of the 2PB as these parts of the cell moved closer to the ZP. Similar events were seen in the opposite half of the zygote around the first indent.

### Emerging two-cell stage

The two vector vortex patterns were intermittently seen in each cell for up to 30 mins after the two-indents stage and their vorticity sign and direction of rotation between the two daughter cells exhibited mirror symmetry (Albrecht-Buehler, 1977). These patterns were not seen in every frame at this early stage, and as the two-cell stage progressed they were increasingly rare and of lower magnitude (Fig. 4K-L). To substitute for their intermittent appearance, vorticity was calculated using a method in which both concentric circles of vectors and incomplete vector patterns could be measured.

### Two-cell stage cytoplasm flow

Each cell of the two-cell stage had vorticity measured from 100 mins after the two-indents stage to the end of each video. These records were interrupted when the embryo was rotating rapidly or the daughter cells grossly overlapped (see above section on zygote acrobatics during and after cytokinesis), and embryos were only scored when the vorticity sign differed significantly between the pair of cells (Chi-squared test). At low temporal and spatial resolution, it was common to find that one of the two earlier vortices dominated the vorticity of each whole cell. In 15/18 two-cell stages, the vorticity sign in one whole cell differed between the cell pair (Chi square <0.05). When it was possible to determine the vorticity sign in relation to 2PB position then the vorticity sign of whole cells showed movement towards the 2PB position (8/10 embryos). This represented the flow of cytoplasm on either side of the cell boundary towards the 2PB, clockwise in one cell and anti-clockwise in the other cell and in two embryos these differences persisted for up to 550-650 mins after the two-indents stage. These observations suggested that the kinetic organization of the daughter cells could be inherited from the parental cell for long periods.

At higher spatial resolution, it was clear that there were significant vorticity differences between the two ends of each cell. Each cell was by eye divided in half across its longest axis, and vorticity was measured in each half in 100 mins periods, with the earliest period starting between 30-99 mins after the two-indents stage. Thirteen out of 17 embryos showed significant differences between the vorticity sign of each half cell in at least one cell in at least one of these periods. The vorticity sign of each end of a cell reversed between two adjacent 100 mins periods in two out of 10 embryos, and in a further two of 10 embryos the rotation direction differed between each end of the cell during the 550-650 mins period after the two-indents stage of division. The conclusion was that cell cytoplasm represented by its grey scale image could rotate in different directions in two halves of the cell for at least 10 h after the two-indents stage but that at this resolution it appeared to be a rare event after the initial 100 mins period (data not shown).

The present results were sufficient to demonstrate that mouse vorticity was either unusual or more carefully analysed than in any other vertebrate because the ends of the cells differed in the direction of cytoplasmic flow for long periods and the direction could also reverse. A speculation is that similar vortices at the two-cell stage will be found when it is searched for in other animal phyla.

## DISCUSSION

### What is the function of the shape changes?

Some explanation is needed for the evolution of the numerous changes in sub-zona space that has been observed (see Introduction, this study, Fig. 5). It is a speculation that these movements improve the vigour and vitality of the zygote and two-cell stage. The problems of demonstrating such an exchange function include the lack of inhibitors that only supress movement and ignorance about rate limiting exchanges in the reproductive tract (Leese, 2003). Despite these difficulties, it is a reasonable idea that these movements have an exchange function because *in vitro* the zygote is surrounded by gradients of oxygen and metabolites (Houghton et al., 1996; Ottosen et al., 2007; Trimarchi et al., 2000a,b). Certainly, the cell number and subsequent *in vivo* development of the pre-implantation stages are improved by increasing medium flow past zona-enclosed zygotes (Heo et al., 2010), and higher rates of oxygen consumption are associated with the vitality of the four-cell stage (Ottosen et al., 2007).

### What are the functions of cytoplasm circulation?

Cytoplasm circulation is well known in other cell types where they promote transport of organelles: examples include the recirculation of the insulin-like growth factor II mannose-6-phosphate receptor, actin flow in motile cells, and axonal transport by known motors. The flows observed here could be considered as no more than a by-product of cytokinesis because cytoplasm must move on either side of the elongating spindle as cytoplasm flows into the two emerging cells, examples in Rappaport, 1966. These vortices may improve exchange between the cell and its periphery by simply stirring up the cytoplasm or they might indicate the movements of specific transport molecules. A further speculation is that two-cell stage may retain a memory of these early vortices and that their direction may influence the next division.

### Summary

The cell membrane and cytoplasm frequently changed their form during the first embryonic cell cycle and the early two-cell stage. The zygote's shape changes were usually repeated sequences of rounder–distorted–rounder, and these sequences occurred inside the zona pellucida, which usually retained its asymmetric shape: consequently, the morphokinetics altered the sub-zona space by as much as 14.8 μm in local regions. The two-cell stage pitched, rotated and probably rolled inside the ZP, again displacing sub-zona fluid. At division to the two-cell stage and well into the following interphase, there was counter-rotating vorticity at each end of the cell and these within cell differences of flow could persist well into the two-cell stage. A plausible function for these changes of shape and cytoplasm movements was to promote exchange with the environment. Similar sequences and two-cell movements were seen in a small sample of human embryos.

## MATERIALS & METHODS

### Mice and media

All experimental procedures applied to animals were approved by the Home Office (UK) or by the Local Ethical Committee no. 1 (Warsaw, Poland), and were performed in compliance with the national regulations. Eight-to 12-week-old mice [CD1, Charles Rivers or F1 (C57Bl6/Tar×CBA/Tar), Faculty of Biology, University of Warsaw] were super-ovulated with an intraperitoneal injection of 8-12 IU of pregnant mare serum gonadotrophin (Intervet) followed 48 h later by 10 IU of human chorionic gonadotrophin (Intervet), and each placed with a single male. Copulation plugs identified mated females the next day and they were killed by cervical dislocation between 22 and 28 h post HCG (hpHCG). The zygotes were freed from the oviduct by dissection in M2 medium (Quinn et al., 1982) at RT, and the subsequent filming conditions can be found in the video recording section. After filming, the zygotes were rinsed and cultured on in KSOM medium with or without amino acids (Summers et al., 2000). The medium was homemade or from reconstituted powder (Millipore, UK, Ltd).

The report of CD1 zygote movements was limited to those that subsequently developed into morulae or blastocysts by 96 h post chorionic gonadotrophin (hpHCG) injection for superovulation, while the F_1_C57BL6/CBA x F_1_C57BL6/CBA (F2) zygotes were filmed under conditions in which parallel studies had shown that *c*. 90% developed into blastocysts (Milewski et al., 2018). Although shape changes occurred in both types of zygotes, their extent was greatest in the CD1 zygotes and the bulk of the analysis was based on this stock.

### Inhibitors

The F2 zygote films started at 25-26 hpHCG and lasted for 15-16 h. One sample was untreated and cultured in KSOM alone. Another sample was preincubated for 30 mins in 30 µM BAPTA-AM in KSOM and maintained in the same medium for filming. The last sample was preincubated in 2 µg/µl cytochalasin D in KSOM for 30 mins and filmed in the same medium.

### Egg restraint

The embryos were filmed in 35 mm diameter MaTek dishes. To stop the embryos from moving about in the recording frame, two methods were used. In Warsaw, F2 zygotes were in a small drop of medium (20 µl), while in Oxford the CD1 embryos were in pens of punched silicone sheet. The most convenient sheet was unwashed Silpuran (gift of Wacker, UK Ltd). Rectangles (c. 4×4 mm) were cut out of 300- or 100-µm-thick sheet, and irregular holes 200-1000 µm wide punched near the centre with the sawn off and sharpened flat ends of 17-19 G syringe needles. The holes were tapered towards their base by placing the thin sheet on 2 mm thick silicone rubber before using the punch. The pens were held on the bottom of the dish during their further sterilization with 100 µl of 70% (v/v) aqueous propanol and dried out for 1-2 h at 37°C. Alternatively, a mesh was used (Booth et al., 2007). In both cases, a glass ring baffle (OD 15 mm, ID 11 mm, height 5 mm) was placed around the restraint and held in place with low melting point paraffin wax. The ring was part filled with 300 µl of KSOM, which was next covered, with 150 µl of mineral oil (Sigma-Aldrich), and the rest of the dish covered with 1 ml of KSOM, topped with 2.3 ml of oil.

### Video recording

Images were collected in Warsaw and Oxford under similar conditions and zygotes were placed in equilibrated KSOM under mineral oil and imaged under an inverted microscope (Zeiss Axiovert) at 37.5°C under 5%CO2 in 95% air (v/v). Further conditions in Warsaw were as follows: high-resolution bright field images (without DIC) from a single equatorial plane were collected by a camera attached to the microscope (Zeiss AxioCam HRm) through a 20× objective (Zeiss Plan Neofluar 20×/0.5) every 2 mins (light source: a halogen lamp Hal 100 set at 1.5 V, intermittent illumination and image exposure time, 4 ms, binning 1×1, image resolution with a co-site sampling: 2600×2060 pix). On the captured image, the resolution was approximately 0.26 µm per pixel.

The conditions in Oxford differed in the following respects: excluded from study was the first 30-60 mins of culture when the zygote shrunk. This reaction of CD1 zygotes was taken to result from transfer from M2 medium at RT to KSOM medium at 37°C. The zygotes were in pens (above). Illumination was intermittent at 1- or 2-min intervals with a CoolLED 635 nm or 770 nm light source (4 ms nominal illumination and camera exposure, 2-5% power), and images collected with a Zeiss objective (LD A-Plan 0.3 NA Ph) followed by either ×0.63 or ×1.0 projection lenses (Hamamatsu ORCA 05G camera, collected at 8-bit detail). The magnification of the captured image was slightly less than 0.5 µm per pixel. When F2 and CD1 images were analysed manually with ImageJ (Schneider et al., 2012), then the captured images were enlarged and represented as 0.21 µm per pixel.

### Human embryo collection

Human embryos were donated from patients attending the Oxford Fertility Clinic with approval from the Human Fertilization and Embryology Authority (centre 0035, project RO198) and the Oxfordshire Research Ethics Committee (Reference number 14/SC/0011). Informed consent was attained from all patients.

### Human embryo culture

Egg restraint and video recording conditions were the same as those for mouse embryo culture in Oxford with the following exceptions. Cook's cleavage medium was used for culture and the recording interval ranged from every 10 s to every 30 mins depending on the circumstances. At the end of the culture period, the material was fixed in 5% paraformaldehyde in PBS solution A (Dulbecco and Vogt, 1954).

### Computational image analysis

Two computational image analysis tools were used. The first was an automatic tool for identifying the outline of the cell membrane in each of the time series images. This tool was based on the method of Alexopoulos et al. (Alexopoulos et al., 2002) using sobel edge detection and dynamic programming to identify the cell outline. The method was tailored to suit the time series images in this study by allowing a user defined weighting to be applied that biased the outline for each image based on the outline identified in the previous time series image. Smoothing factors could also be defined depending on the imaging conditions to reduce the high frequency noise in the outline.

The second tool was used to quantify the motion of the cytoplasm within the cell. This tool was based on image analysis techniques used in particle image velocimetry (PIV) an approach commonly used in fluid dynamics. The method is based on identifying matching sub-regions within sequential pairs of images in time and has been used previously in related work (Ajduk et al., 2011). Normalized cross-correlation was used to match square interrogation windows between images with successively reducing window sizes. The initial and final interrogation windows were usually in the ratio 64:32 pixels but on rare occasions when image noise was sufficiently small the ratio was 64:16 pixels.

These tools were accessed via a front menu, and the group of programs was named ‘cell-piv’. For the software and manual contact Dr Shane Windsor (Shane.Windsor@bristol.ac.uk).

The outline function of the image analysis software rarely exactly followed cell profile at maximum elongation in division and the two-cell stage. The last points on elongation axis and the emerging two-cell axis were measured through visual CoA with ImageJ (Schneider et al., 2012).

## Supplementary Material

Supplementary information
