# Supplementary material for: Dynamic shapes of the zygote and two-cell mouse and human
Source: Biol Open. 2021 Dec 22;10(12):bio059013. doi: 10.1242/bio.059013 (PMC8713988; doi:10.1242/bio.059013)
Supplement: Supplementary information [file biolopen-10-059013-s1.pdf]

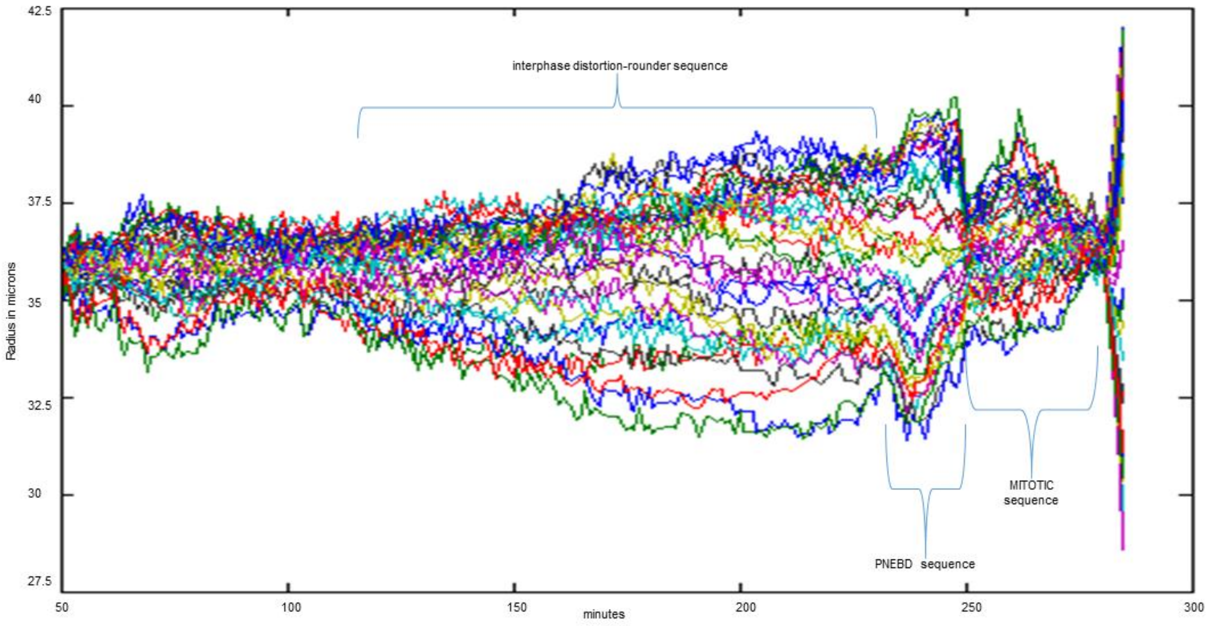

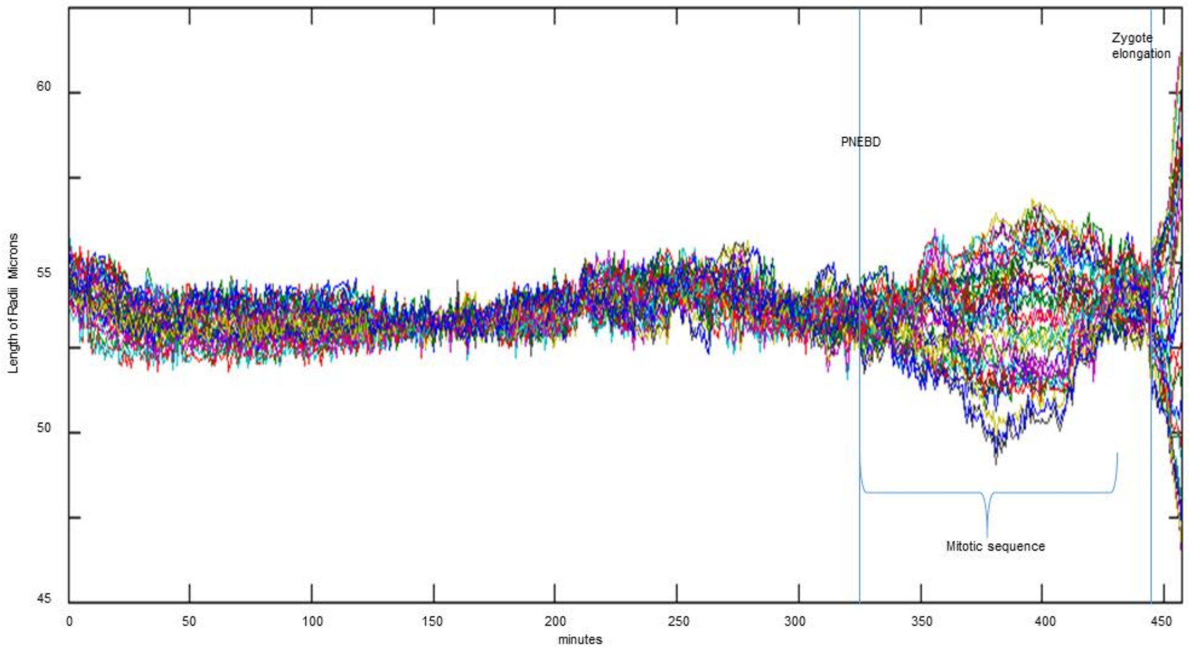

Fig S1. Mouse and Human rounder-distortion-rounder sequences

A. Mouse: The cell_piv software displayed all 36 radii at 10° rotation (below). Each sequence showed a smaller range of radial lengths when the shape was rounder and a larger range of lengths when the zygote was distorted (see text Figs 1 & 2).

B. Human: This human egg did not have a PNEBD rounder-distortion-rounder sequence of sufficient magnitude to meet the criteria established in the text. The mitotic sequence stood out in this radial display.

Biology Open • Supplementary information

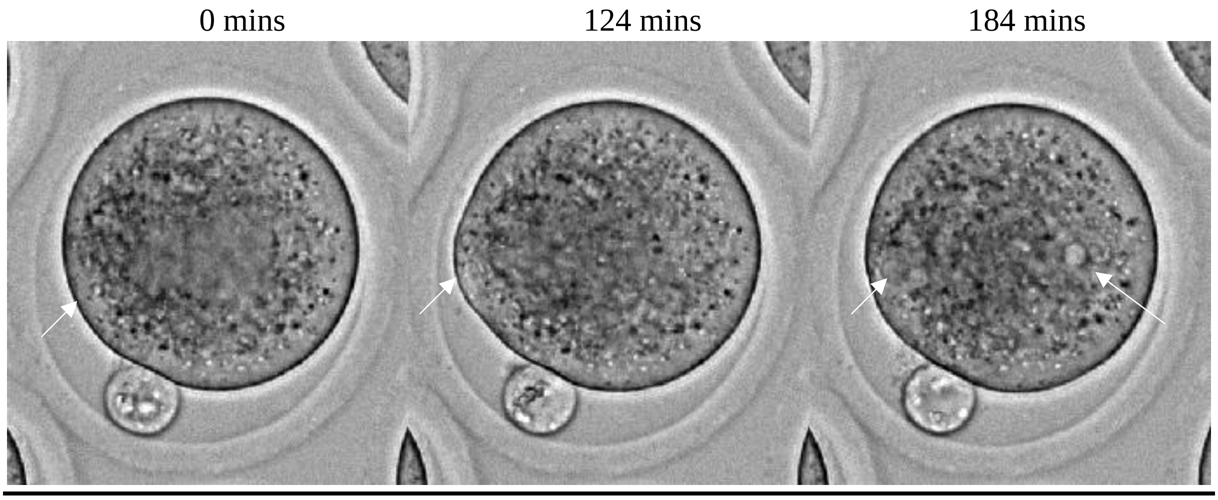

Fig S2. Mouse: Surface bulge formation and retraction in cytochalasin treated zygotes. The membrane bulges appeared to be induced by dispersal of the contents of a nucleus (small dots near zygote membrane in the first images at the start of each row). At dispersal, it was not known if the chromatin was in micronuclei or free of any membrane. Later at 184 mins, the dispersed nuclear material was gathered up in a single nuclear membrane.

Biology Open • Supplementary information

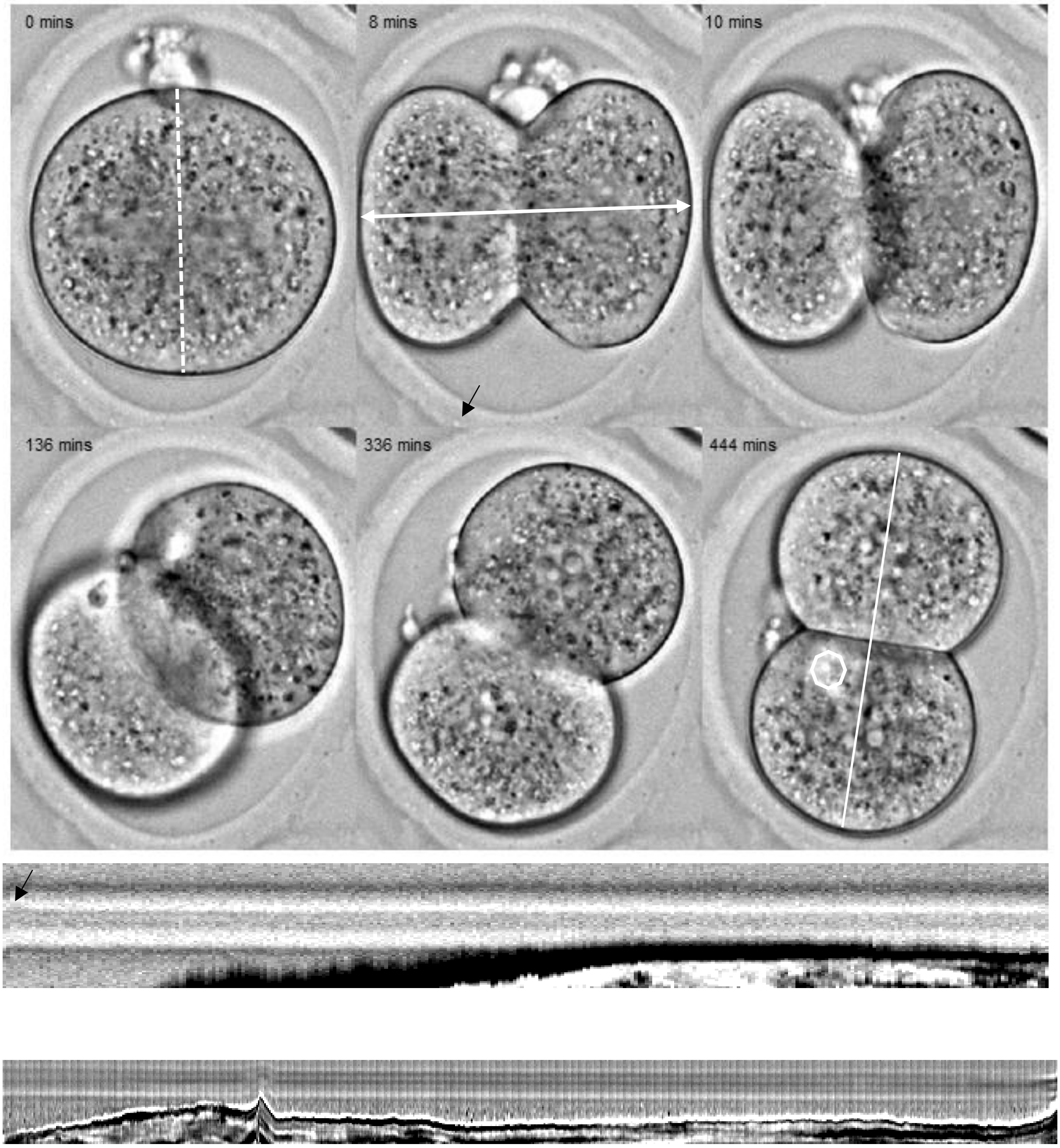

Biology Open • Supplementary information

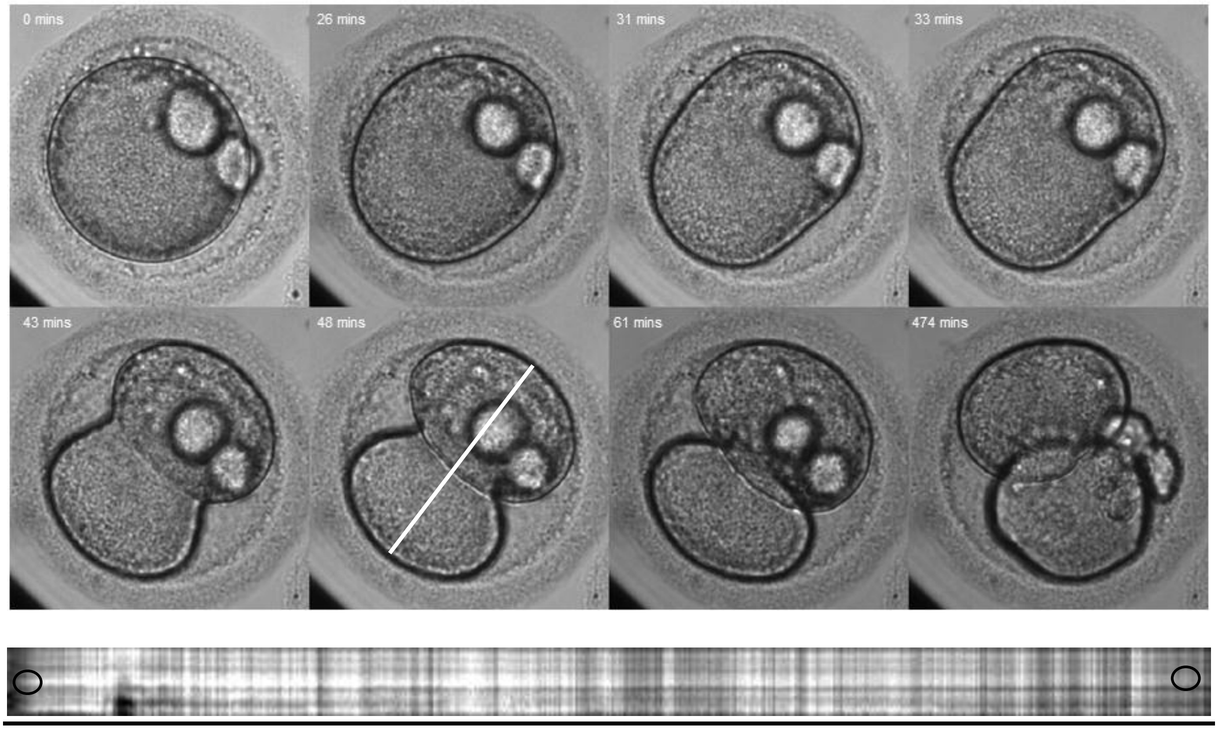

Fig S3. Pitch, Rotate, and Roll in Mouse and Human

Mouse: The evidence for pitching comes from the focus of each ends of the elongating zygote and of the pair of cells at the 2-cell stages.
0-10 mins: The left cell started to move out of focus while the elongating nascent 2-cell axis (arrowed white line) was perpendicular to the narrower diameters of the ZP.
136-444 mins: The images of the two cells overlapped (136 mins), and they came into the same level of focus as the 2-cell axis rotated into the longer diameters of the ZP (444 mins). The 2-cell axis (thin white line 444 mins) nearly coincided with the 2PB diameter at 0 mins (dotted white line).
The 2PB became incorporated into the outline of the 2-cell stage where it could just be discerned (444 mins, white circle). The 2PB repositioning was interpreted as the 2-cell stage rolling around its axis. The ZP changed shape between 0 and 8 mins as the sub-zona space was occluded at the ends of the elongating axis of the zygote (line with two arrowheads). The ZP was resliced at 8 mins to give the kymograph in the bottom panel. In both panels, the black arrow indicated the white dot that appeared as a white streak in the kymograph. The black crescent in the kymograph is the outline of the bottom left cell as the 2-cell stage rotated. It does not distort the ZP.

Human: Pitch, Roll and Rotate: As the zygote elongated (26 mins), so its bottom left part went out of focus, and it became overlapped, by the top right cell at 61 mins (pitching). The 2-cell axis (white line) rotated during this 8 hour record but this rotation could not be measured because the 2-cell axis was oblique at the end of the record. At 477 mins the polar bodies had emerged from the 2-cell profile as if the embryo had rolled around the 2-cell axis. The ZP moved slightly and a white dot (circled) came into the 1 pixel thick slice shortly after the start of the kymograph (left) and began to fade towards the end of the recording at 500 mins. The intensity of illumination varied (vertical lines on kymograph).

Biology Open • Supplementary information
